# Evaluation of Sciatic Nerve Stiffness Using Shear Wave Elastography in Patients with Unilateral Diabetic Foot Ulcers

**DOI:** 10.3390/diagnostics13030547

**Published:** 2023-02-02

**Authors:** Shun-Ping Chen, Ting-Ting Ye, Jing Hong, Hong Zhu

**Affiliations:** 1 Department of Ultrasonography, First Affiliated Hospital of Wenzhou Medical University, Wenzhou 325000, China; 2 Department of Endocrinology, First Affiliated Hospital of Wenzhou Medical University, Wenzhou 325000, China

**Keywords:** diabetic foot ulcer, diabetic peripheral neuropathy, sciatic nerve, ultrasound, elastography, shear wave elastography

## Abstract

Objective: To evaluate the stiffness of the sciatic nerve by shear wave elastography (SWE) and to determine whether SWE can be used to predict diabetic foot ulcer (DFU) in a patient with diabetic peripheral neuropathy (DPN). Methods: Sixteen patients (thirty-two lower limbs) with unilateral DFU were studied retrospectively. The ultrasonographic parameters including cross-sectional area (CSA) of sciatic nerve, intraneural blood flow, peak systolic velocity (Vmax) and resistive index (RI) in the intraneural artery of the sciatic nerve, and the SWE stiffness value of the sciatic nerve were measured. The examinations of arteries of the lower limbs were also performed by ultrasound. According to the presence or absence of DFU, the 32 lower limbs were divided into two groups: the DFU group and the non-DFU group. The ultrasonographic parameters were compared between these two groups. Results: There was no significant difference (*p* > 0.05) between the two groups for CSA, intraneural blood flow, Vmax and RI in the intraneural artery of the sciatic nerve, and numbers of severe artery stenosis or full occlusion of the artery in the lower limbs. However, SWE stiffness values in the sciatic nerve in the DFU group are higher than the non-DFU group (*p* < 0.05). When the SWE stiffness values were used for prediction of DFU in patients with DPN, the area under the ROC curve (AUC) was 0.727 (95% CI: 0.541–0.868). When the best SWE stiffness value of 24.48 kPa was taken as a cutoff for prediction of DFU, the sensitivity was 62.50% (95% CI: 35.4–84.8%), and the specificity was 75% (95% CI: 47.6–92.7%). Conclusions: Sciatic nerve stiffness is significantly higher in lower limbs with DFU. SWE is a noninvasive imaging method that may be used to evaluate sciatic nerve stiffness, then potentially predict DFU in patients with DPN.

## 1. Introduction

Diabetes is one of the most common diseases and its incidence is growing rapidly, as seen by the exponential increase in its global prevalence over the last 30 years [1]. Diabetic foot ulcer (DFU) is the most common complication associated with diabetes [2]. The prevalence of DFUs is 4–10% in the diabetic population [3]. The risk of patients with diabetes developing DFU across their lifetime has been estimated to be 19–34% [4]. DFUs frequently become infected, which may result in some type of lower extremity amputation in 20% of DFUs [5]. Additionally, DFU is a major and one of the most expensive causes of hospital admissions among diabetes patients, accounting for 20–40% of resources allocated for diabetes [6]. The common risk factors for DFUs are diabetic peripheral neuropathy (DPN), peripheral arterial disease, trauma, and foot deformities. Among these risk factors for DFUs, DPN is the most common and accounts for 86% of cases [7]. Therefore, early diagnosis of DPN and early prediction of advanced DPN (which can develop into serious complications such as DFUs), would benefit most diabetic patients with timely treatment, and lead to delay or prevention of progression to advanced DPN. Consequently, this will reduce the cost of treatment and improve the quality of life of diabetic patients.

Generally, electrophysiological examination is a gold standard for the diagnosis of DPN; however, this technique is limited by its invasiveness, cost, and discomfort [8]. Therefore, clinical examination became a primary diagnosis of DPN. Both methods have disadvantages with respect to the evaluation of large nerve fibers such as sciatic nerves affected by DPN, which may indicate advanced DPN. In recent years, imaging techniques such as magnetic resonance imaging (MRI) and ultrasound (US) are emerging for the diagnosis of DPN [9,10]. Because it is noninvasive, US has become a good complementary tool for the diagnosis of DPN. In a previous study [10], enlarged nerve cross-sectional area (CSA), increased intraneural blood flow, and echo changes of the peripheral nerve were reported to be associated with DPN. More recently, a new US technology, shear wave elastography (SWE), which is able to measure the stiffness or elasticity of tissues, reflected in the elastic changes in the degree of tissue deformation after compression, was used for the evaluation of nerve stiffness and prediction of DPN [8,11,12,13,14]. The study [14] showed that SWE not only can be used for diagnosis of typical DPN, but also has a potential use for cases with clinically defined DPN. However, the relationship between sonoelastographic nerve stiffness in the lower limb and DFU in patients with DPN has not been reported. We hypothesized that sonoelastographic stiffness (SWE stiffness value) of the lower limb sciatic nerve is associated with DFU in diabetic patients with DPN. Thus, the aim of this study was to investigate the relationship between sonoelastographic stiffness (SWE stiffness value) of the lower limb sciatic nerve in the presence and absence of DFUs. The unilateral lower limbs with DFUs were taken as the study group, while the contralateral lower limbs (without DFUs) were taken as the control group.

## 2. Methods

### 2.1. Patients

**Figure 1:**
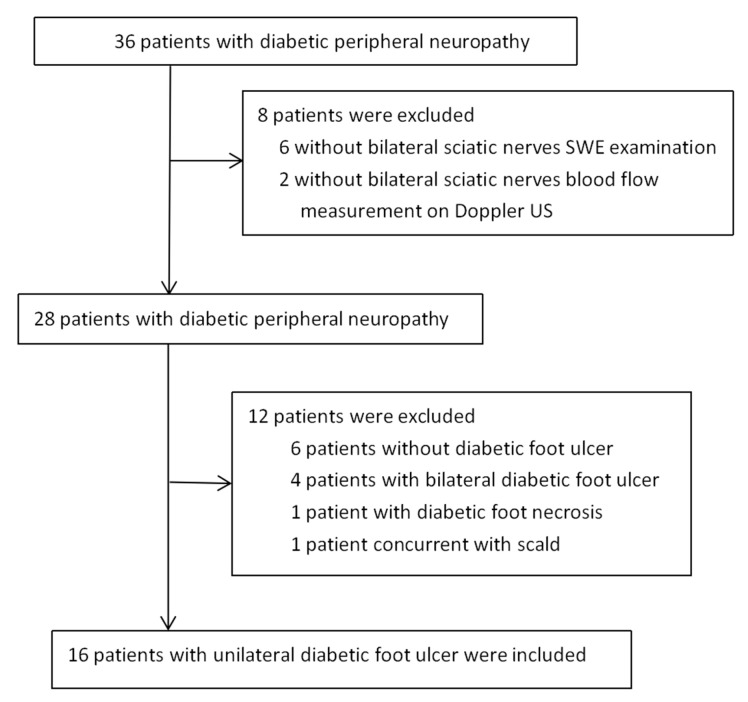
Flowchart of the process for selecting patients with unilateral diabetic foot ulcer.

This retrospective self-controlled study was approved by our institutional review board (No. 2021-R027). Patients diagnosed with diabetic foot on the lower limbs by nerve US examination from June 2018 to July 2019 were retrospectively studied. The requirement for informed consent was waived. A total of 36 consecutive cases were reviewed. Patients were included if: (a) they had type 2 DFUs in a unilateral lower limb; (b) they had DPN confirmed by electromyogram or clinical findings or both; (c) they underwent sciatic nerve US examination (including gray-scale, Doppler and elastography US); and (d) they also underwent vessels US including venous and artery examination of the lower limb, and the time between vessels US and elastographic US examination was within one week. Patients were excluded if they had trauma or foot deformities. Body mass index was calculated using the following formula: weight in kilograms divided by the square of height in meters. There were 16 patients (32 lower limbs) included in this retrospective study. Figure 1 presents the patient selection flowchart for this study.

Of those 16 patients with DPN, 15 patients were diagnosed by both electromyogram and clinical findings, and one patient was diagnosed by clinical findings. According to the lower limb with or without unilateral DFU, the lower limbs were classified as the DFU (target) group and the non-DFU (contralateral or control) group. Demographic data, clinical history, and outcomes were obtained from the hospital’s electronic medical information database.

Type 2 diabetes mellitus was diagnosed by using the World Health Organization (WHO) criteria, which includes fasting plasma glucose level ≥126 mg/dL (7.0 mmol/L) or 2-h postprandial plasma glucose level ≥200 mg/dL (11.1 mmol/L) during OGTT or a random plasma glucose ≥200 mg/dL (11.1 mmol/L) if the patient presents classic symptoms of hyperglycemia or hyperglycemic crisis. The clinical criteria for DPN included the following: more than one symptom (foot pain, weakness, numbness, tingling, or ataxia) or sign (abnormal knee or ankle reflexes, temperature, light touch, monofilament, or vibration sensation). 

Electromyography (key-point, Alpine Biomed ApS, Denmark) was used to evaluate the motor nerve conduction velocity (MCV) of the ulnar, median, tibial, and common peroneal nerves and their sensory branches, and the sensory nerve conduction velocity (SCV) of sural and superficial peroneal nerves. The reference values were in accordance with the results provided by Tang [15]. The cutoff value for diagnosing the slowed nerve conduction velocity (NCV) was below 20% of the control NCV. The electrophysiologic criteria for the diagnosis of DPN was having one or more nerves with conduction abnormality in a patient. 

### 2.2. Ultrasound (US)

All US examinations (multiple-mode ultrasound included gray-scale, Doppler, and elastography ultrasound) were performed in the ultrasonographic department at The First Affiliated Hospital of Wenzhou Medical University, Wenzhou, China. US examination of the sciatic nerve was performed on subjects in a prone position with the knee extended and hip in a neutral position. All images of the sciatic nerve were acquired by a sonographist with 6 years of experience in musculoskeletal US. A Logiq E9 (GE Healthcare, Wauwatosa, WI, USA) US system operating at a 9 MHz high-frequency (for SWE measure, the length of the transducer is 5 cm) and a 6–15 MHz high-frequency (for CSA and blood flow observation and measurement, the length of the transducer is 6 cm) linear array transducer were used in this study. 

**Figure 2:**
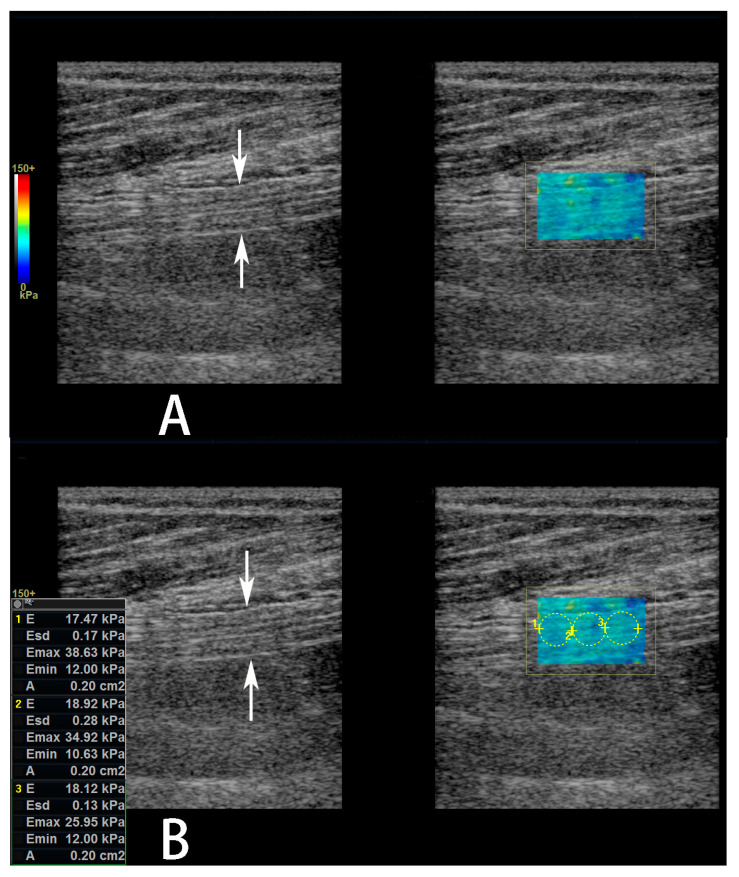
Measurement of sciatic nerve shear wave elastography (SWE) stiffness value. (**A**,**B**) Gray scale images (**left** panel) of the sciatic nerve and its affiliated color-coded SWE images (**right** panel) in a 61-year-old female diabetic patient with diabetic peripheral neuropathy (DPN). In the left panel, the sciatic nerve is shown between arrows. In the right panel, a color-coded box is superimposed on the image to indicate the sciatic nerve. (**B**) In the right panel, three non-overlapping circular regions of interest have been superimposed on the 2-D shear elasticity map that covered as much of the sciatic nerve as possible. The average SWE stiffness values within each region of interest (ROI) has been automatically calculated and shown on the screen.

The sonographic parameters of the sciatic nerve were measured at about 3 cm proximal to the bifurcation of the tibial and common fibular nerves. Initially, transverse imaging of the bifurcation of the tibial and common fibular nerves were obtained, and the bifurcation positioned in the centre of the screen. Then, the transducer was rotated 90^0^ to obtain longitudinal imaging of the nerve. Next, the transducer was moved towards the proximal thigh and the bifurcation disappeared on the screen. Lastly, the transducer was re-rotated 90^0^ to obtain transverse imaging of the sciatic nerve. First, the CSA of the nerve was measured on the transverse image using a manual trace directly over the epineurium. Then, power Doppler US was performed in both transverse and longitudinal imaging planes (a parallel orientation to the sciatic nerve fibers) to observe the blood flow within the nerve. If the intraneural blood flow existed, then the spectral Doppler US was performed on the brightest vessels to observe the wave form and measure the velocity (for arteries, the peak systolic velocity (Vmax) and the minimal diastolic velocity (Vmin) were measured) and resistive index (for arteries, RI). The RI was defined as [1 − (Vmin ÷ Vmax)]. Last, SWE was performed in the longitudinal imaging plane. A color-coded box was superimposed on the image, and the size of the acquisition box was kept the same (20 × 14 mm) for all the patients. The spectrum scale of colors ranged from blue for softer tissues to red for stiffer tissues. When the frozen images of the nerves were obtained (probe was held stationary for 3 s), a circular region of interest (ROI) with a diameter of 2–4 mm was placed within the nerve according to the nerve’s diameter to represent the SWE stiffness value measurements. The mean elasticity within the ROI are expressed as the mean of three different measurements, and was reported in kiloPascals (kPa) (Figure 2). 

Additionally, the US examinations of arteries in the lower limbs were performed within one week before or after the sciatic nerve US examination according to the reference criteria [16]. The diagnosis of severe artery stenosis (70–99% stenosis) in the lower limbs was defined as flow velocity ratios between the stenosis site and proximal corresponding artery greater than or equal to 4 [16,17]. The diagnosis of a full occlusive artery in the lower limbs was defined as detecting no flow signal in the corresponding artery by duplex Doppler.

The default machine parameters (musculoskeletal setting) of the gray-scale imaging and power Doppler were applied. When the power Doppler was used, the gain was increased until aliasing from the surrounding soft tissues was abolished [18]. The SWE range was set from 0 to 150 kPa utilizing a musculoskeletal setting. All US images of the sciatic nerve were assessed for technical quality prior to analysis. Images were analyzed and stored in the Logiq E9 hard drive. According to the presence or absence of DFU in a unilateral lower limb, the lower limbs were classified as DFU (target) and non-DFU (contralateral or control) groups.

### 2.3. Statistical Analysis

The MedCalc statistical software (version 12.0; Frank Schoonjans, Mariakerke, Belgium) was used for statistical analysis. Statistical significance between compared groups was estimated using Fisher’s exact tests for categorical variables and Student’s *t*-tests for continuous variables. Correlations between compared parameters were made using Pearson’s correlation coefficients. Results were considered significant for *p* < 0.05. The receiver operating characteristic (ROC) curve for the parameter (SWE stiffness value) was constructed to obtain the best cutoff value for prediction of DFU. The best cutoff was correlated with the highest accuracy resulting from the maximal Youden index.

## 3. Results

### 3.1. Study Demographics

The clinical characteristics of the patients are shown in Table 1. Of the sixteen patients, nine patients were men and the remaining seven were women (average age: 64.4 ± 7.1 y [range: 54–76 years]). From the 16 patients with DFUs in unilateral lower limbs, 11 had DFUs in the left lower limb. The duration of diabetes and of DFUs were 11.9 ± 7.6 years (range: 5–30 years) and 17.5 days (range: 10.0–30.0 days), respectively. 

The symptoms in the affected lower limbs were pain (*n* = 7), or numbness (*n* = 3), or both (*n* = 4), or without pain and numbness (*n* = 2). According to Wagner’s grade for the ulcers, there were nine, four, and three cases in grade II, III, and IV, respectively. All ankle reflexes disappeared. The body mass index for the 16 patients was 24 ± 2.2 (range: 19.1–28.4). 

### 3.2. Measurements Using Conventional and Doppler US

The US findings on the sciatic nerves in two groups are listed in Table 2. There was no significant difference (*p* > 0.05) between the two groups for CSA, intraneural blood flow, as well as arteries’s V_max_ and RI of the sciatic nerve. 

Table 3 shows artery finding on color Doppler in the lower limbs. There was no significant difference (*p* > 0.05) between the two groups for numbers of severe artery stenosis or full occlusion of the artery in the lower limb. The duration between vessels US and elastographic US examination was 0–3 days.

### 3.3. Measurement of Sciatic Nerve Stiffness and Assessment of Its Relationship with DFUs

**Figure 3:**
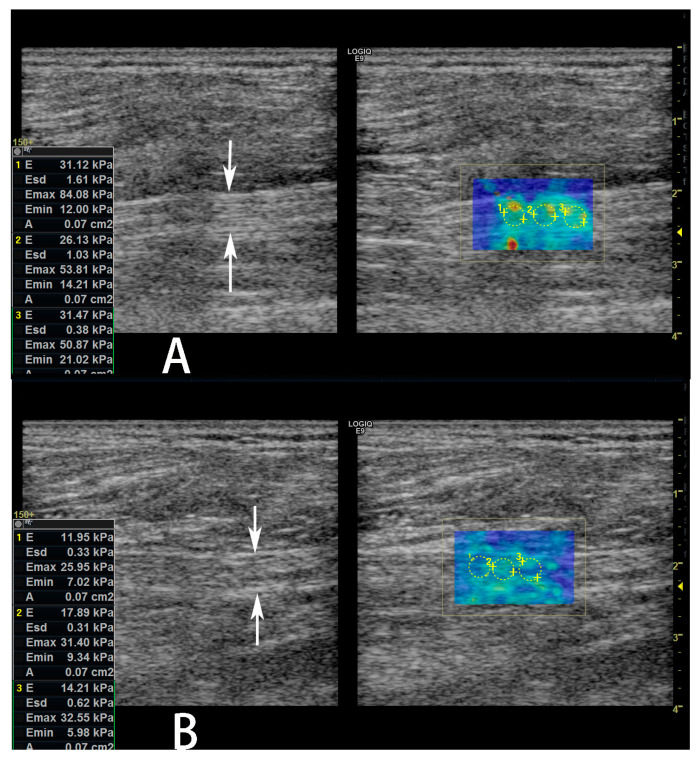
Shear wave elastography (SWE) images of the sciatic nerve in a 61-year-old male diabetic patient with diabetic peripheral neuropathy (DPN). He also had a diabetic foot ulcer (DFU) on the left lower limb. The average SWE stiffness value of the left sciatic nerve was 29.57 kPa (**A**), which was higher than SWE stiffness value of the sciatic nerve, average 14.68 kPa; (**B**) the right lower limb without DFU.

**Figure 4:**
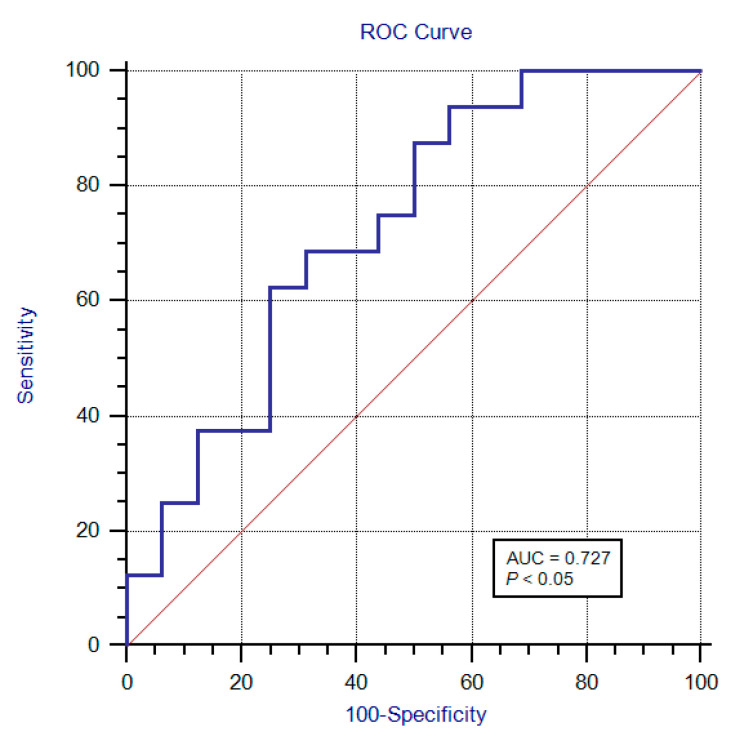
The area under the ROC curve (AUC) was 0.727 (95% confidence interval [CI]: 0.541–0.868) when the SWE values were used for prediction of DFU in patients with DPN. When the best cutoff value of SWE was 24.48 kPa for prediction of DFU, the sensitivity and specificity were 62.50% (95% CI: 35.4–84.8%) and 75% (95% CI: 47.6–92.7%), respectively.

A significant difference (*p* < 0.05, Table 2 and Figure 3) was found between the elastographic (SWE) stiffness values of the sciatic nerves of the two groups. Namely, SWE stiffness values in the sciatic nerve in the DFU group are higher than the non-DFU group. When the SWE values were used for prediction of DFU in patients with DPN, the area under the ROC curve (AUC) was 0.727 (95% confidence interval [CI]: 0.541–0.868, Figure 4). When we used the best SWE value of 24.48 kPa as a cutoff for prediction of DFU, the sensitivity and specificity were 62.50% (95% CI: 35.4–84.8%), and 75% (95% CI: 47.6–92.7%), respectively. When we used the SWE values of 10.45 kPa and 42.8 kPa as cutoff for prediction of DFU, the sensitivities were 100% (95% CI: 79.4–100.0%) and 12.50% (95% CI: 1.6–38.3%), respectively, while the specificities were 31.25% (95% CI:11.0–58.7%) and 100.0% (95% CI: 79.4–100.0%), respectively. However, there was no significant correlation between the SWE stiffness values of the sciatic nerves in the lower limbs with DFU and Wagner’s grade for the ulcers (r = 0.151, *p* = 0.576).

## 4. Discussion

DFUs are a major health problem, often leading to lower limb amputations and increased death rates [5], and consequently increasing social and medical burdens. The early prediction of DFUs in patients with DPN may benefit diabetes patients. In previous reports, various methods such as lower limb sensory testing, thermography, and peak plantar pressure assessment and commonly available clinical and laboratory data were used for predicting DFUs [19,20]. To assess whether US could be used to predict advanced DPN in diabetes patients, we hypothesized that US, particularly SWE, can be used for predicting DFUs in patients with DPN.

### 4.1. Conventional and Doppler US Findings

Few reports are available on the use of nerve CSA for the evaluation of sciatic nerve lesions in diabetic patients [21]. A previous study using MRI showed that the sciatic nerve was larger in patients with painful diabetic polyneuropathy than in those without pain symptoms [21]. However, in our study, no significant difference was found between the two groups when the CSA of the sciatic nerves were compared. 

The intraneural blood flow is another feature for evaluating the nerve of the diabetic patients with DPN [10]. The cause for intraneural blood flow in diabetic patients has been attributed to hypervascularity of the epineural vessels in response to endoneurial ischemia secondary to microangiopathy [22]. In our study, we found intraneural blood flow in 81% (26/32) of the sciatic nerves of patients with DPN, which is higher than previous report [22] that diabetic nerves had 28% of nerve blood flow detection by power Doppler. Then, the spectral Doppler US further confirmed that the intraneural blood flow in the sciatic nerves in our study not only came from the artery but also from the vein. To the best of our knowledge, this has not been reported in previous studies. However, no significant difference was observed between the two study groups for the presence of intraneural blood flow, as well as for the V_max_ or RI in the arteries. The explanation for this requires further investigation.

DPN is the most important risk factor for DFUs, while peripheral arterial disease is the second risk factor for DFUs [6]. In our study, we found no significant difference between the two groups with or without DFU for concurrent peripheral arterial disease including arterial severe stenosis and/or occlusion. Therefore, in our study, peripheral arterial disease does not seem to be a determining factor for DFU.

### 4.2. SWE Stiffness Value of Sciatic Nerves and Its Relationship with DFUs

Our findings showed that lower limbs with DFU had higher SWE values of the sciatic nerves than contralateral lower limbs without DFU in patients with DPN (*p* < 0.05). A previous study showed that the sciatic nerve stiffness can be assessed accurately using SWE, and the stiffness of the sciatic nerve can be affected by the lower limb movements [23]. Wang et al. [24] used SWE to demonstrate that the longer the duration of the unilateral lumbar disc herniation, the greater the stiffness of the sciatic nerve. However, in our study, we found that the greater the stiffness of the sciatic nerve in patients with DPN, the greater the possibility for DFU to occur. To the best of our knowledge, this has not been reported in previous studies.

We can suggest some probable mechanisms that explain why the patients with DFU had greater stiffness in their sciatic nerves than the patients with DPN but without DFU. The perineurium is the connective tissue surrounding the sciatic nerve fascicles. It is mechanically strong but less flexible. It remains under constant pressure by maintaining intraneural tissue pressure. In patients with DPN, the edema within the sciatic nerve fascicle increases intraneural pressure, making the nerve stiffer [11]. Furthermore, the degree of edema within the sciatic nerve fascicle would be more severe in the patient with DFU than those with DPN but without DFU, which would make the sciatic nerve stiffer. These hypotheses require further confirmation.

Our study showed that, in patients with clinically and (or) electrophysiologically established DPN, the sciatic nerve stiffness was exceedingly higher in the lower limbs with DFU than contralateral lower limbs without DFU. A cutoff value of 24.48 kPa revealed high sensitivity and specificity in the prediction of DFU. This finding has important clinical significances in that SWE may be used to evaluate sciatic nerve stiffness to potentially predict advanced DPN or early DFU with DPN, and then may guide clinical treatment strategy. Additionally, the finding also may be used for follow-up of patients with DFU during treatment.

### 4.3. Strengths and Limitations

The study had some strengths. First, although a lower limb ulcer is associated with lots of factors in addition to DFN, such as cardiovascular diseases which is common in patients with diabetes, the study was designed as a self-controlled study which may minimize our results affected by some confounding factors. Second, though our results showed that only SWE stiffness value associated with DFU, ultrasounds were performed for study of the sciatic nerve in multiple-mode ultrasound manners (gray-scale, Doppler, and elastography ultrasound). Third, all examinations and analysis of the sciatic nerve ultrasound were performed by an experienced sonographer, which may maximize observer agreement.

This study also had some limitations. First, this was a retrospective, single-institution study, with the risk of potential bias with respect to data collection. Second, DPN and DFU often involve multiple nerves, but we measured only the sciatic nerve; thus, there is a need to study multiple nerves simultaneously in further studies. Third, although no significant difference was observed between the two study groups with respect to peripheral arterial disease in the lower limbs, we confirmed peripheral arterial disease using Doppler US, and not with digital subtraction angiography. Finally, the size of the patient cohort in our study was small, which may cause bias. However, to our knowledge, this study is the first study to date to provide a new imaging method (SWE) for evaluation of the sciatic nerve to predict DFU. As a pioneer, our study shows the feasibility of SWE in predicting DFU in patients with DPN, and provides a new possibility for noninvasive evaluation of DFU. However, further multicenter retrospective or prospective studies with a greater number of patients are needed to determine the effectiveness of using SWE to evaluate the sciatic nerve for predicting DFU in patients with DPN.

## 5. Conclusions

Sciatic nerve stiffness is significantly higher in lower limbs with DFU. SWE is a noninvasive imaging method that may be used to evaluate sciatic nerve stiffness to potentially predict DFU in patients with DPN. This finding possibly demonstrates that the SWE stiffness value increased in large nerves, except sciatic nerves, in patients with unilateral DFUs. Further investigations are needed to identify which large nerves, except sciatic nerves, have increased stiffness and determine the number of large nerves that would be involved in patients with unilateral DFUs. Moreover, SWE of large nerves together with commonly available clinical and laboratory data should be used in the future to predict DFUs in patients with DPN.

## Figures and Tables

**Table 1:** Clinical characteristics of 16 patients with unilateral diabetic foot ulcers.

Characteristic	Patients
Male (%)	9 (56.3%)
Age (years)	64.4 ± 7.1
BMI (kg/m^2^)	24 ± 2.2
Smoking (%)	5 (31.3%)
Alcohol use (%)	2 (12.5%)
Diabetes duration (years)	11.9 ± 7.6
Diabetic foot ulcer duration (days)	17.5 (10.0–30.0)
Wagner classification (%)	
2	9 (56.3%)
3	4 (25.0%)
4	3 (28.7%)
Hypertension (%)	14 (87.5%)
Atherosclerosis (%)	16 (100%)
Hyperlipidemia (%)	1 (6.3%)
Coronary heart disease (%)	1 (6.3%)
Diabetic retinopathy (%)	9 (56.3%)
Diabetic nephropathy (%)	10 (62.5%)
Creatinine (μmol/L)	91.0 (62.0–217.3)
ALB (g/L)	34.1 ± 5.7
Hb (g/L)	110.1 ± 22.8
HbA1c (%)	8.3 (7.0–9.1)
TC (mmol/L)	3.91 (3.07–4.69)
TG (mmol/L)	1.05 (0.71–1.80)
HDL-C (mmol/L)	0.99 ± 0.21
LDL-C (mmol/L)	1.96 (1.36–2.61)

BMI: body mass index; ALB: albumin; Hb: hemoglobin; HbA1c: hemoglobin A1c; TC: total cholesterol; TG: triglyceride; HDL: high density lipoprotein; LDL: low density lipoprotein.

**Table 2:** Ultrasound findings of the sciatic nerve in 16 patients with unilateral diabetic foot ulcers.

US Parameters	DFU Group	Non-DFU Group	*p* Value
SN area(cm^2^)	0.526 ± 0.188	0.519 ± 0.204	0.832
SN with Flow (*n*)	14	12	0.695
SN flow velocity (cm/s) *	13.160 ± 14.063	5.460 ± 3.903	0.066
SN flow RI *	0.909 ± 0.184	1	0.152
SN stiffness values (KPa)	28.660 ± 10.485	19.435 ± 10.183	0.002

DFU: diabetic foot ulcer; SN: sciatic nerve; RI: resistance index; US: ultrasound. * 24 SNs with intraneural blood flow in bilateral limbs, and two SNs with intraneural venous blood flow in two patients confirmed by spectral Doppler were excluded. Thus, 20 SNs with intraneural arterial blood flow in bilateral limbs were compared.

**Table 3:** Ultrasound findings of the lower limb artery severe stenosis or occlusion in 16 patients with unilateral diabetic foot ulcers.

Artery (*n*)	DFU Group	Non-DFU Group	*p* Value
Common femoral artery	0	1	0.317
Superficial femoral artery	3	0	0.083
Popliteal artery	2	0	0.157
Arteriae tibialis anterior	6	5	0.480
Posterior tibial artery	3	3	1.000
Total	14	9	0.297

DFU: diabetic foot ulcer.

## Data Availability

Research data are available on request from the corresponding author.
